# *Antidesma thwaitesianum* Müll. Arg. Fruit Juice, Its Phytochemical Contents, Antimicrobial Activity, and Application in Chiffon Cake

**DOI:** 10.1155/2022/5183562

**Published:** 2022-01-28

**Authors:** Patcharaporn Tinchan, Arpassorn Sirijariyawat, Arunya Prommakool, Kriangkrai Phattayakorn, Soukbandith Pheungsomphane, Chintana Tayuan

**Affiliations:** ^1^Kasetsart University, Kamphaeng Saen Campus, Faculty of Agriculture, Nakhon Pathom Province, Thailand; ^2^Kasetsart University, Chalermphrakiat Sakon Nakhon Province Campus, Faculty of Natural Resources and Agro-Industry, Sakon Nakhon Province, Thailand

## Abstract

*Antidesma thwaitesianum* Müll. Arg. or Mao is well-known in Thailand for its use in soft drinks and wine. This study investigated the potential of its fruit juice as a food preservative based on its antimicrobial activity against food-borne pathogens and spoilage. The fruit juice had antibacterial activity against *Bacillus cereus* TISTR1527, *Staphylococcus aureus* TISTR2329, *Listeria monocytogenes* DMST17303, *Pseudomonas aeruginosa* TISTR357, *Salmonella* Typhimurium DMST562, and *Escherichia coli* TISTR074. The minimum inhibitory concentration ranged from 25 to 100 mg/ml. Of significance was that the juice had excellent antifungal activities against *Aspergillus flavus* TISTR3135 and *Penicillium digitatum* ATCC10030. Furthermore, the fruit juice increased the lag time of spore germination of *P. digitatum*. Analysis of the phytochemical contents of the juice showed that the total phenolic and flavonoid contents were 20.07 mg GAE/g and 3.57 mg QE/g, respectively. In antioxidant activity assay, the juice exhibited moderate DPPH and ABTS^+^ radical scavenging and ferric-reducing activities. The addition of 5% fruit juice extended the microbial shelf life of chiffon cake. The treated chiffon cake had a shelf-life of 7 days, compared to 3 days for the untreated control. These results support the possible use of Mao fruit juice as an antimicrobial agent and a natural food preservative.

## 1. Introduction

*Antidesma thwaitesianum* Müll. Arg., known as “Mao” in Thai, is in the family Phyllanthaceae which is most diverse in South-East Asia. It is a tropical fruit commonly found in Northeast Thailand [1]. Almost all parts of this plant are used for the treatment of various disease due to having cytotoxic, antidiabetic, antioxidant, antiradical, thrombolytic, antiplatelet, anticoagulant, antidysenteric, antimicrobial, antihypertensive, anticancer, and sudorific activity [2]. Mao fruit is well-known in Thailand for its use in soft drinks, wine, and healthy foods [3, 4]. Ripe fruit is rich in 5-hydroxymethylfurfural (HMF), several volatile compounds, organic acid, long-chain fatty acid, and photochromic compound [5]. Mao fruits have also been reported to contain phytochemicals that have great antioxidant potential [6] and provide various biological activities [2, 7]. However, the various parts of Mao fruit have differences in their phytochemical compounds. Whole fruits have confirmed the presence of phenolic compounds [4]. Furthermore, anthocyanin, flavonoids, and phenolic acid were present in the methanolic fruit extracts [8]. The main polyphenolic components in the fruits are gallic acid, (-)-epicatechin, (+)-catechin, and cyanidin-3-O-glucoside [6]. Seeds and marcs are abundant sources of polyphenols and proanthocyanidins [9]. While the fruit juice contains high amounts of several bioactive components such as phenolics, ascorbic acid, anthocyanins, and flavonoids [10–12]. Mao fruit extracts exhibited antimicrobial properties [13]. However, only antibacterial activity of Mao fruit extracts against a limited number of pathogens was reported [3, 4]. There is a lack of reporting on the antimicrobial potential of the fruit juice against food-related microorganisms, especially spoilage molds. This research is aimed at evaluating the antibacterial activities of Mao fruit juice against foodborne pathogens and food spoilage microorganisms. In this work, we are reporting, for the first time, on antifungal activity of Mao fruit juice. In addition, the phytochemical contents and antioxidant activity of the fruit juice and its application in food systems were investigated. To our knowledge, no investigation has addressed the use of Mao fruit juice as a natural preservative in chiffon cake.

## 2. Materials and Methods

### 2.1. Test Microorganisms

Six bacterial strains and two mold strains were used in this experiment. *Pseudomonas aeruginosa* TISTR357, *Escherichia coli* TISTR074, *Salmonella* Typhimurium DMST562, *Listeria monocytogenes* DMST17303, *Staphylococcus aureus* TISTR2329, *Bacillus cereus* TISTR1527, and *Aspergillus flavus* TISTR3135 were obtained from Thailand Institute of Scientific and Technological Research (TISTR) cultures, and Department of Medical Sciences Thailand (DMST culture collection)*. Penicillium digitatum* ATCC10030 was obtained from the American Type Culture Collection (ATCC, USA).

### 2.2. Plant Material and Preparation

The Fapratan cultivar of *A. thwaitesianum* Müll. Arg. was used in this study. Fresh Mao fruits were collected from Phu Phan municipality, Sakon Nakhon province, Thailand. The fruits were washed and squeezed, and the marc and seed were separated. Their juice extracts were dried using a lyophilizer at -55°C under vacuum.

### 2.3. Antimicrobial Activity Screening

The Mao juice was screened for its antimicrobial activity against foodborne pathogens and spoilage microorganisms using the agar well diffusion method as described by Al-Zoreky [14] with slight modification. Bacterial suspension at a concentration of 10^7^ CFU/ml was inoculated on Mueller-Hinton agar (MHA) using a pour plate technique. Mold spores at a concentration of 10^5^ spores/ml were inoculated on potato dextrose agar (PDA) plates. Wells (6 mm diameter) were punched into the inoculated plates using a sterile cork borer. Each well was filled with 100 *μ*l of the juice at a concentration of 200 mg/ml. Gentamycin and amphotericin B were used as positive controls for the analysis against bacteria and mold, respectively. The bacterial cultures were incubated at 37°C for 24 h, while the mold cultures were incubated for 5 days at 25°C for *Penicillium digitatum* and at 30°C for *Aspergillus flavus*. Antimicrobial activity was detected by measuring the zone of inhibition (including the well diameter) that appeared after the incubation.

### 2.4. Determination of Minimum Inhibitory Concentrations

The minimum inhibitory concentration (MIC) is defined as the lowest concentration of an antimicrobial agent that will inhibit the visible growth of a microorganism. The MIC of Mao juice was tested using the agar diffusion method as described by Gonelimali et al. [15]. The bacterial suspension and mold spores were inoculated on MHA and PDA, respectively, using the pour plate technique as previously described. Wells (6 mm diameter) were cut into the inoculated agar. Different concentrations of juice at 12.5, 25, 50, 100, or 200 mg/ml were prepared using two-fold serial dilution. Samples (each 100 *μ*l) of the juice were transferred to the respective wells. Gentamycin and amphotericin B were used as positive controls, with sterile water serving as the negative control. Then, each plate was incubated as described above. The growth of the respective microorganisms was observed. The MIC was considered as the lowest concentration that inhibited the visible growth of the organisms.

### 2.5. Determination of Minimum Bactericidal and Fungicidal Concentrations

Two lowest concentrations of the juice exhibiting invisible growth from MIC plates were tested for minimum bactericidal and fungicidal concentrations (MBC and MFC, respectively) using a broth dilution technique. Bacterial inoculum (100 *μ*l) of 10^7^ CFU/ml or spore suspensions of 10^5^ spores/ml were mixed with 200 *μ*l of antimicrobial juice to produce a final inoculum of approximately 10^6^ CFU/ml of bacterial cultures or 10^4^ spores/ml of mold spores. After 16-18 h, the tested suspensions were spread on agar (MHA or PDA) plates and incubated as previously described. The concentration of the juice causing negative growth of bacteria and mold was considered as the MBC and MFC values, respectively [16].

### 2.6. Determination of Spore Germination

Spore germination of *P. digitatum* and *A. flavus* was determined following a previously described procedure with modification [17]. *P. digitatum* and *A. flavus* were grown on PDA plates and incubated at 25°C and 30°C, respectively, for 5 days. Spores from each test mold were suspended in 10 ml of sterile water with 5% glycerol and diluted to provide spore concentrations of 10^5^ spores/ml. The spore suspensions were inoculated into PDA at a final spore concentration of 10^4^ spores/ml. PDA with the mold spores was poured over PDA supplemented with Mao juice at concentrations of 50, 100, or 200 mg/ml. Controls were prepared without fruit juice. PDA inoculated with *P. digitatum* was then incubated at 25°C while *A. flavus* was incubated at 30°C for 5 days. Periodically, three agar discs were aseptically cut from each agar plate using a sterile cork borer. The discs were placed on a glass slide where spore germination was examined microscopically and considered positive when at least 10% of the spores had germinated. The lag phase for germination was the number of hours needed for 10% of the spores to germinate.

### 2.7. Determination of Total Phenolic Content

Determination of total phenolic content (TPC) was performed in 96-well microplates using Folin-Ciocalteau's method according to Hansakul et al. [18]. A sample (30 *μ*l) of fruit juice, 150 *μ*l of Folin-Ciocalteau's reagent, and 120 *μ*l of Na_2_CO_3_ were added to each well. The plate was incubated at room temperature for 30 min. The absorbance was measured at 765 nm. The total phenolic content was expressed as milligrams of gallic acid equivalents per gram of dry juice (mg GAE/g).

### 2.8. Determination of Total Flavonoid Content

The total flavonoid content (TFC) was measured using aluminum chloride colorimetric assay as reported by Poontawee et al. [19]. A sample (20 *μ*l) of fruit juice was added in 96-well microplates. Ethanol (60 *μ*l), AlCl3 (10 *μ*l, 10%), potassium acetate (10 *μ*l, 1 M), and distilled water (120 *μ*l) were mixed and added to each well. The plate was incubated at 37°C for 30 min in the dark. The absorbance was determined at 415 nm. The total flavonoid content was expressed as milligrams of quercetin equivalents per gram (mg QE/g).

### 2.9. Ferric-Reducing Antioxidant Power Assay (FRAP)

The reducing capacity of the juice was determined using a ferric-reducing antioxidant power assay (FRAP) assay as described by Hansakul et al. [18]. To prepare the FRAP solution, 300 mM of acetate buffer (pH 3.6), 20 mM of ferric chloride hexahydrate, and 10 mM of TPTZ in 40 mM of HCl were mixed in a ratio of 10 : 1 : 1. A sample (20 *μ*l) of fruit juice at a concentration of 100 *μ*g/ml was added to 96-well microplates followed by FRAP solution (180 *μ*l). The plate was incubated at room temperature for 30 min. The absorbance was measured at 590 nm. The ferric-reducing ability was determined from a standard curve of iron (II) sulfate solution and expressed as FRAP (mg Fe^2+^/g) and TEAC values (mg TE/g).

### 2.10. DPPH Radical Scavenging Assay

Antioxidant activity was determined using a modified version of the method described by Puangpronpitag et al. [9]. A sample (100 *μ*l) of fruit juice at a concentration of 100 *μ*g/ml was added to a 96-well microtiter plate. Then, 300 *μ*l of 0.1 mM 2, 2-diphenyl-2-picrylhydrazyl (DPPH) in ethanol solution was added to each well. The plate was incubated at 37°C for 30 min in the dark. Absorbance was measured at 517 nm on a microplate reader. Trolox was used as the positive control, and distilled water was used as the blank. The scavenging activity of the juice against DPPH radicals was expressed as the half maximal effective concentration (EC50; *μ*g/ml) and as trolox equivalent antioxidant capacity (TEAC) values (mg TE/g).

### 2.11. ABTS^+^ Radical Scavenging Activity Assay

Antioxidant activity was determined using ABTS^+^ assay as described by Seebaluck-Sandoram et al. [20]. ABTS radical cations (ABTS^+^) were generated by reacting a 7 mM 2,2′-azino-bis (3-ethylbenzothiazoline-6-sulfonic acid diammonium salt (ABTS) stock solution with 2.45 mM potassium persulfate and keeping overnight in the dark to yield a dark blue solution. The ABTS^+^ radical solution was diluted with ethanol to an absorbance of 0.70 to 0.90 ± 0.02 at 734 nm. A sample (10 *μ*l) of fruit juice at a concentration of 100 *μ*g/ml was added to 190 *μ*l of ABTS cation radical solution in a 96-well microplate. The plate was incubated at room temperature for 6 min in the dark. The absorbance was measure at 734 nm. The scavenging activity of the extracts against ABTS^+^ radicals was expressed as EC50 (*μ*g/ml) and TEAC values (mg TE/g).

### 2.12. Antimicrobial Activity of Mao Juice in Chiffon Cake

Chiffon cake was prepared using the following ingredients: cake flour, vegetable oil, baking soda, water, sugar, salt, eggs, and potassium tartrate. Five percent of Mao juice was added into the ingredients. After baking at 175°C for 20 min, the chiffon cake was placed on a cooling rack until ambient temperature was reached. Then, cake samples were placed in sterile polyethylene bags and stored at 4°C. The total viable count (TVC) and yeast and mold counts were analyzed at 1, 3, 5, 7 and 9 days of storage. Twenty-five g of sample were mixed with 225 ml of 0.1% sterile Butterfield's phosphate-buffered dilution water. Decimal dilutions were prepared of 10^−2^, 10^−3^, 10^−4^, and others as appropriate by transferring 10 ml of the previous dilution to 90 ml of diluent. Appropriate dilutions were pour plated in plate count agar (PCA) for the determination of TVC. For yeast and mold counts, samples were spread plated in Dichloran 18% glycerol (DG18). Then, the PCA and DG18 plates were incubated at 35°C for 24 hours and at 30°C for 5 days, respectively. The microbial counts of chiffon with Mao juice were compared to the control that had no added juice.

### 2.13. Statistical Analysis

The experiment was performed in triplicate. Results are reported as mean values ± standard deviation. Microbiological data were transformed into logarithms, expressed as log CFU/g.

## 3. Results and Discussion

### 3.1. Phytochemical Contents and Antioxidant Activity

The extraction yield of Mao juice was 15.23% (Table 1). Phytochemical analysis of the Mao juice showed the presence of phenolic compounds and flavonoids. The total phenolic and flavonoid contents were 20.07 mg GAE/g and 3.57 mg QE/g, respectively. These results were in line with another report where the total phenolic content of Mao fruit juice was 22.67 mg GAE/g [3]. There has been little reporting of the flavonoid content in Mao juice; however, Chinprahast et al. [21] found that total flavonoid contents in Mao fruit extracts with different solvents ranged from 18.45 to 255.16 mg QE/100 g. Krongyut and Sutthanut [22] also reported that the ethanolic extract of Mao ripe fruits, yield 12.08%, contained phenolics (11.6 mg GAE/g extract) and flavonoids (0.30 QE/g extract). Butkhup and Samappito [8] reported that gallic acid and (+)-catechin were the main phenolic acid and flavonoid, respectively, found in Mao berries. Three complementary tests (DPPH, ABTS^+^ free radical-scavenging activity, and reducing power assay) were used to assess the antioxidant activity in the Mao juice, and the results are presented in Table 2. The juice had radical scavenging activity against both DPPH radicals (EC50 = 72.18 *μ*g/ml) and ABTS^+^ radicals (EC50 = 76.95 *μ*g/ml). The FRAP value was 184.59 mg Fe^2+^/g. The juice had moderate antioxidant power compared to the positive standard trolox. Antioxidant activity is significantly correlated with the phenolic compounds found in a plant [6, 20], and the antioxidant activity of phenolic compounds is attributed to their donating electron, scavenging free radicals, and reducing power [23]. The antioxidant activity of fruit juices is the synergistic effect of different antioxidants existing in the juice [24]. Hardinasinta et al. [12] reported that Mao juice has relatively high antioxidant activity, and significant correlation was found between the antioxidant capacity and some polyphenolic compounds. The antioxidant activity of Mao fruits is related to gallic acid, ferulic acid, and some anthocyanins, mainly cyanidin-3-O-glucoside [6].

### 3.2. Determination of Antimicrobial Activity

The antimicrobial activities of Mao juice were tested against different food spoilage organisms and foodborne pathogens. Mao juice had antibacterial activity against all bacterial strains, namely, three strains of Gram-positive (*B. cereus*, *S. aureus*, and *L. monocytogenes*) and three strains of Gram-negative (S. Typhimurium, *P. aeruginosa*, and *E. coli*) (as shown in Table 3). *L. monocytogenes* was the most sensitive to the Mao juice with an inhibition zone of 26.97 mm. The MIC for *L. monocytogenes* was 25 mg/ml, with a higher MIC of 50 mg/ml for *B. cereus*, *S. aureus*, S. Typhimurium, and *E. coli*. The antibacterial activity of plant extracts has been attributed to individual phenolic compounds [25–27]. Plants containing phenols, tannins, and flavonoids are effective against bacterial species [28]. The biological activity is related to their molecular structure (hydroxyl group or phenolic ring) resulting in phenolic compounds having the capacity to attach proteins and bacterial membrane to form a complex [29]. Phenolic compounds have been reported to have varying antimicrobial activity against foodborne pathogens. The current study showed that Gram-positive bacteria were more susceptible to Mao juice than Gram-negative bacteria. Other researchers have also suggested that natural antimicrobial substances, such as essential oils and extracts were more active against Gram-positive bacteria than Gram-negative bacteria [30, 31]. Notably, the Mao juice had antifungal activity against *A. flavus* and *P. digitatum*, with inhibition zone diameters of 13.65 and 24.24 mm, respectively, and MIC value of 50 mg/ml. Antifungal growth was observed as a clear circular zone of inhibition. Furthermore, in the current study, Mao juice retarded the germination of *P. digitatum* conidia. Mold spores in the control agar germinated after 32 h of incubation at 25°C, while no germination occurred in the presence of the fruit juice at concentrations of 50-200 mg/ml (Table 4). Spore germination of *P. digitatum* was observed in the test with fruit juice after 72 h of incubation. The lag phase for germination of *P. digitatum* increased from 24 hour to 48 hours. Fungal strains differ in their susceptibility to plant extract [32], as shown in the current study where Mao fruit juice was unable to inhibit spore germination of *A. flavus*. Natural extracts have different effects on fungal growth. Mohammedi and Atik [33] reported that extracts from *D. gnidium* exhibited complete inhibitory effect on mycelium growth and spore germination of *A. flavus*, while *H. scoparium* exhibited inhibitory effect only on spore germination. Fresh leaf extracts of *Flourensia cernua* caused reduction in the colony growth of *Alternaria alternata*, *P. digitatum*, and *Colletrotichum gloeosporiodes*; however, the extracts had no effect on the sporulation [34]. The antifungal activity of plants has been related to bioactive compounds with the principal groups being alkaloids, anthraquinones, flavonoids, and tannins [35]. The microcidal effect was also examined in this study; however, the juice had no bactericidal and fungicidal activity against any of the tested microorganisms. Nonetheless, the results showed that Mao juice had strong antibacterial and antifungal properties. This observation differed somewhat from Dechayont et al. [3] who, using a disc diffusion method, reported that the juice at a concentration of 5 mg per disc had no antimicrobial activity against *S. aureus*, *E. coli*, or *S.* Typhimurium. Differences between the studies may have been related to many experimental variances such as concentrations of the extracts, bacterial strains, and the methodology applied for assay. Similar negative results were reported for other fruit juices such as cranberry, with Côté et al. [36] reporting that cranberry juice had no antibacterial effect against *L. monocytogenes* HPB, *E. coli* O157:H7 EDL 933, *E. coli* ATCC 25922, 2812, *S.* Typhimurium SL1344, *S. aureus*, and *P. aeruginosa* ATCC 15442. In contrast, Harich et al. [37] found that cranberry juice had antibacterial activity against Gram-positive *L. monocytogenes* and the Gram-negative pathogenic bacteria *S. typhimurium* and *E. coli* O157:H7. In the current study, Mao juice did not have any microcidal activity against any of the tested bacteria and molds, suggesting that perhaps a higher concentration is required to achieve mortality. Fatmawati et al. [38] also reported that the antimicrobial effect increased with an increase in the concentration of the fruit extract. For this reason, Mao juice in the concentration range 25-200 mg/ml is supposed to be a microstatic antimicrobial against both bacteria and mold.

### 3.3. Application of Mao Juice in Chiffon Cake

Bakery products are intermediate moisture foods and are mainly spoiled by yeasts and filamentous fungi. Saeed et al. [39] reported that *A. niger*, *A. flavus*, *A. fumigatus*, *A. terreus*, *Alternaria alternata*, *Curvularia americana*, *Fusarium solani*, *P. digitatum*, *Saccharomyces cerevisiae*, and *Geotrichum candidum* were found in bakery samples. In the current study, the efficacy of the fruit juice in controlling microbial growth in chiffon cake was examined for 9 days at 4°C storage. Results showed that all samples had increasing TVCs during refrigerated storage (Table 5). However, TVC was lower than the value of 4.0 log CFU/g, which was considered as the upper microbiological limit for bakery products, as defined by the Ministry of Public Health (Thailand). However, yeast and mold counts were higher than the permissible limit of 2.0 log CFU/g on day 5 for untreated chiffon cake. The results showed that the addition of 5% fruit juice significantly inhibited the growth of yeast and mold in a baked product. The shelf-life of treated sample was extended to 7 days that was over double the 3 days for untreated chiffon cake. The study demonstrated the potential application of Mao juice as an ingredient in chiffon cake to inhibit yeast and mold. The results indicate its potential as a natural food preservative in a baked product.

## 4. Conclusions

This study revealed that *Antidesma thwaitesianum* Müll. Arg. (Mao) fruit juice had antimicrobial activity against food spoilage organisms and food borne pathogens. The present study is the first report on the antifungal activity of Mao fruit juice against *Aspergillus flavus* TISTR3135 and *Penicillium digitatum*ATCC10030. Mao fruit juice contains bioactive compounds and demonstrated antioxidant capacity. The antimicrobial efficacy of Mao fruit juice showed that it could be used as a natural promising preservative, which could extend the shelf life of a bakery product to 7 days.

## Figures and Tables

**Table 1:** Extraction yield, total phenolic content (TPC), and flavonoid content (TFC) of Mao fruit juice.

Extraction yield (%)	TPC (mg GAE/g)	TFC (mg QE/g)
15.23 ± 0.81	20.07 ± 3.43	3.57 ± 0.96

Values are shown as mean ± standard deviation (*n* = 3). GAE: gallic acid equivalent; QE: quercetin equivalent.

**Table 2:** Antioxidant activity of Mao fruit juice determined using DPPH, ABTS^+^, and FRAP testing.

Sample	DPPH scavenging activity		ABTS^+^ scavenging activity		Ferric-reducing antioxidant power	
	EC_50_ (*μ*g/ml)	TEAC value (mg TE/g)	EC_50_ (*μ*g/ml)	TEAC value (mg TE/g)	FRAP value (mg Fe^2+^/g)	TEAC value (mg TE/g)
Juice	72.18 ± 1.90	475.11 ± 12.52	76.95 ± 3.66	569.90 ± 26.57	184.59 ± 3.44	90.59 ± 1.69
Trolox	34.26 ± 0.55	—	43.76 ± 0.82	—	203.76 ± 5.44	—

Values are shown as mean ± standard deviation (*n* = 3). DPPH: 2, 2-diphenyl-2-picrylhydrazyl; ABTS: 2,2′-azino-bis (3-ethylbenzothiazoline-6-sulfonic acid) diammonium salt; FRAP: ferric-reducing antioxidant power; EC_50_: half maximal effective concentration; TEAC: trolox equivalent antioxidant capacity; TE: trolox equivalent.

**Table 3:** Inhibition zones related to MIC using different Mao fruit juice concentrations.

Juice (mg/ml)	Inhibition zone (mm)							
	*B. cereus*	*S. aureus*	*L. monocytogenes*	*E. coli*	*P. aeruginosa*	*S. Typhimurium*	*A. flavus*	*P. digitatum*
25	0.00 ± 0.00	0.00 ± 0.00	9.55 ± 0.48	0.00 ± 0.00	0.00 ± 0.00	0.00 ± 0.00	0.00 ± 0.00	0.00 ± 0.00
50	6.29 ± 0.26	6.03 ± 0.02	21.55 ± 0.48	11.65 ± 1.02	0.00 ± 0.00	11.42 ± 1.02	10.31 ± 1.36	16.58 ± 0.59
100	8.73 ± 0.64	7.03 ± 0.43	24.83 ± 0.45	17.61 ± 1.19	9.36 ± 1.28	18.61 ± 0.67	12.01 ± 1.07	20.43 ± 0.70
200	9.90 ± 0.99	9.48 ± 0.60	26.97 ± 1.52	20.08 ± 1.15	11.41 ± 1.86	21.06 ± 0.80	13.65 ± 0.49	24.24 ± 2.60
Gentamycin	17.52 ± 0.76	16.43 ± 1.59	18.83 ± 1.11	26.05 ± 0.38	11.75 ± 0.93	22.99 ± 0.23	—	—
Amphotericin B	—	—	—	—	—	—	7.99 ± 1.25	10.08 ± 2.31
Juice (mg/ml)	MIC (mg/ml)							
	50	50	25	50	100	50	50	50

**Table 4:** Effect of Mao fruit juice on spore germination of *A. flavus* and *P. digitatum.*

Incubation time (h)	Spore germination							
	Control		50 mg/ml		100 mg/ml		200 mg/ml	
	*A. flavus*	*P. digitatum*	*A. flavus*	*P. digitatum*	*A. flavus*	*P. digitatum*	*A. flavus*	*P. digitatum*
0	—	—	—	—	—	—	—	—
8	—	—	—	—	—	—	—	—
16	+	—	+	—	+	—	+	—
24	+	—	+	—	+	—	+	—
32	+	+	+	—	+	—	+	—
48	+	+	+	—	+	—	+	—
72	+	+	+	+	+	+	+	+
80	+	+	+	+	+	+	+	+

+: at least 10% of mold spores had germinated.

**Table 5:** Microbial count of chiffon cake during storage at 4°C for 9 days.

Samples	Period (days)	Total plate count (log CFU/g)	Yeast and mold count (log CFU/g)
Untreated chiffon cake	1	1.74	1.74
	3	1.30	1.32
	5	2.23	2.40
	7	2.34	2.59
	9	2.60	2.57
Chiffon cake with 5% Mao fruit juice	1	1.58	1.60
	3	2.08	1.00
	5	2.60	1.85
	7	2.81	1.48
	9	3.04	2.23

## Data Availability

All the data relevant to the research are included in the manuscript. Any further information required is available from the corresponding author upon request.
